# Double adverse drug reaction: Recombinant human growth hormone and idiopathic intracranial hypertension - acetazolamide and metabolic acidosis: a case report

**DOI:** 10.4076/1757-1626-2-6534

**Published:** 2009-06-26

**Authors:** Gianluca Tornese, Giorgio Tonini, Federica Patarino, Fulvio Parentin, Federico Marchetti

**Affiliations:** 1 Department of Paediatrics, Institute of Child Health, IRCCS Burlo Garofolo and University of TriesteTriesteItaly; 2 Department of Surgery, Ophthalmology Unit, Institute of Child HealthIRCCS Burlo Garofolo, Trieste, Italy

## Abstract

A 9-year-old girl, treated for growth hormone deficiency, developed bitemporal progressive headache, diplopia, acute comitant esotropia and visual loss 3 months after starting recombinant growth hormone. An increased intracranial pressure was revealed by examination of ocular fundus and lumbar puncture, and the absence of other causes, ruled out through a brain scan, led to the diagnosis of idiopathic intracranial hypertension.

Recombinant growth hormone was discontinued and acetazolamide started up to 30 mg/kg/die without any clinical improvement but developing metabolic acidosis. The switch to intravenous dexamethasone (0.4 mg/kg/die) led to a dramatic clinical improvement after only 1 day, then confirmed by examination of ocular fundus and visual evoked potentials. Currently, there are no evidence-based guidelines for the management of intracranial hypertension, and even though acetazolamide is recognized as the first-line drug, its efficacy and safety have not been proven: some patients might not respond and others will present unacceptable side-effects. Therefore we suggest the use of corticosteroids in intracranial hypertension when acetazolamide is inefficient or intolerable.

## Case presentation

We report a case of a 9-year-old Caucasian Italian girl, treated for growth hormone deficiency (GHD) with recombinant human growth hormone (rhGH) at a total weekly dose of 6 mg. At the diagnosis of GHD her weight was 24.6 kg, her height was 113 cm; physical examination, mental and developmental status were all normal. The diagnosis was made on laboratory criteria (two pathological GH stimulation tests) and on the MRI of the head which excluded brain tumour and anatomical abnormalities.

Three months after starting rhGH, she complained of progressive and bitemporal headaches, nausea, vomiting, diplopia and visual loss. Acute comitant esotropia was the only abnormality in the neurological examination. Ocular fundus examination revealed bilateral papilledema and a lumbar puncture showed an increased intracranial pressure (210 mm H_2_O) with normal cerebrospinal fluid. An MRI scan ruled out intracranial lesions (including venous sinus thrombosis), leading to a final diagnosis of idiopathic intracranial hypertension (IIH) (pseudotumor cerebri).

Since the association between IIH and rhGH treatment has already been described, we promptly discontinued rhGH and started treatment with acetazolamide at the dose of 20 mg/kg/die. Four days after starting acetazolamide, there was no clinical improvement at all, with persistence of headaches, diplopia, nausea, vomiting and weakness. After two more days, despite increasing the dose of acetazolamide up to 30 mg/kg/die, there was no recovery. Indeed, weakness worsened, arterial blood analysis showed metabolic acidosis (pH 7.29; PCO_2_ 31.1; HCO_3_ 15.7; BE - 7.3), likely due to acetazolamide. Therefore we stopped acetazolamide and started the treatment with intravenous dexamethasone (0.4 mg/kg/die). After only one day there was a dramatic clinical response with complete disappearance of headache, progressive improvement in the esotropia and regression of the papilloedema. We decreased intravenous steroids, switching to oral prednisone (1 mg/kg/die) after one week. Ten days after discharge, the girl no longer had headaches nor weakness, and there was no more esotropia or diplopia. Eye examination revealed further improvement of papilloedema. Therapy was tapered down in 3 weeks with no worsening. After two weeks rhGH was restarted at a dose of 1.8 mg weekly, and progressively increased up to 6 mg weekly; the treatment was well tolerated, without any side effects.

## Discussion

rhGH has been increasingly described among those exogene factors responsible for IIH [1-2]. The first cases were reported in 1986, due to the progressive trend in higher and more frequent doses of GH following the introduction of rhGH in 1985. The real incidence of IIH in children treated with rhGH is not known, since IIH itself is a rare event in childhood. According to Australian-New Zealandese OZGROW database, this incidence is 1.2 per 1000 cases overall, but appears to be underestimated [3].

It is thought that GH has an IGF-I-mediated action on choroid plexus, the main site of CSF production, with increased production of CSF and consequent intracranial hypertension. Aggressive GH dosing appears to be a common risk factor in these cases, but the manner in which GH therapy is initiated might be more important than the ultimate dose. It is possible that a gradual dose escalation might eliminate or at least decrease the risk of IIH in children in rhGH therapy, even if the final achieved dose is high.

In the majority of cases IIH straightens out within a few months; however, severe headaches and permanent severe visual impairments represent two possible complications. Both medical and surgical therapies are reported, even though - up to now - the efficacy of treatments for IIH has not been proved: there is insufficient information to generate an evidence-based management strategy for IIH, both in adulthood [4] and for paediatric population [5]. Acetazolamide is largely recognized as the first-line drug [6], but its efficacy for IIH and its safety in childhood have not been proven: some patients might not respond and many Authors report its unacceptable side-effects [7]. Therefore, comparative, randomised, prospective studies to quantify the efficacy and safety of various existing therapeutic options, which are currently widely used in childhood IIH are urgently needed.

In this prospective, the use of corticosteroids should be considered in acute severe cases of IIH when acetazolamide is inefficient or intolerable; even if controversial [6], this strategy is supported - up to now - by single positive case reports [8,9], including our experience.

## Abbreviations

CSF: cerebrospinal fluid
GH: growth hormone
GHD: growth hormone deficiency
IGF-I: insuline-like growth factor-I
IIH: idiopathic intracranial hypertension
MRI: magnetic resonance imaging
rhGH: recombinant human growth hormone
